# The Woman’s Hospital

**Published:** 1881-08

**Authors:** 


The Woman's Hospital.—In another column are given the details of a noble work which has been designed and ably executed in the establishment of a "Woman's Hospital in the State of New York." The origin, growth and accomplishment of this philanthropic undertaking have reached so many in the past, and must effect so great a degree in the future, that its appeal should not be made in vain. It asks for help that it may continue its honorable, dignified and exalted labors for the women of America, and it does not overestimate its claim upon the public when it expresses a hope that its labors may appeal eloquently to the compassion which women should feel for women, and to the sympathy which it is the privilege of the sterner sex to extend to the gentler.

